# Identification and differential expression analysis of MicroRNAs encoded by Tiger Frog Virus in cross-species infection *in vitro*

**DOI:** 10.1186/s12985-016-0530-6

**Published:** 2016-04-30

**Authors:** Ji-Min Yuan, Yong-Shun Chen, Jian He, Shao-Ping Weng, Chang-Jun Guo, Jian-Guo He

**Affiliations:** Guangdong Provincial Key Laboratory of Marine Resources and Coastal Engineering/South China Sea Bio-Resource Exploitation and Utilization Collaborative Innovation Center, School of Marine, Sun Yat-sen University, 135 Xingang Road West, Guangzhou, 510275 PR China; State Key Laboratory for Biocontrol, School of Life Sciences, Sun Yat-sen University, 135 Xingang Road West, Guangzhou, 510275 PR China; Institute of Aquatic Economic Animals and Guangdong Province Key Laboratory for Aquatic Economic Animals, Sun Yat-sen University, 135 Xingang Road West, Guangzhou, 510275 PR China

**Keywords:** *Iridoviridae*, Tiger frog virus, MicroRNA, Cross-species infection

## Abstract

**Background:**

Tiger frog virus (TFV), dsDNA virus of the genus *Ranavirus* and family *Iridoviridae*, causes a high mortality of tiger frog tadpoles cultured in Southern China. MicroRNAs (miRNAs) have been identified in many viruses especially DNA viruses such as Singapore Grouper Iridoviruses (SGIV). MicroRNAs play important roles in regulating gene expression for virus subsistence in host. Considering that TFV infects cells of different species under laboratory conditions, we aim to identify the specific and essential miRNAs expressed in ZF4 and HepG2 cells.

**Methods:**

We identified and predicted novel viral miRNAs in TFV-infected ZF4 and HepG2 cells by deep sequencing and software prediction. Then, we verified and described the expression patterns of TFV-encoded miRNAs by using qRT-PCR and Northern blot.

**Results:**

Deep sequencing predicted 24 novel TFV-encoded miRNAs, and qRT-PCR verified 19 and 23 miRNAs in TFV-infected ZF4 (Group Z) and HepG2 (Group H) cells, respectively. Northern blot was performed to validate eight and five TFV-encoded miRNAs in Groups H and Z, respectively. We compared the expression of TFV-encoded miRNAs from two groups and defined TFV-miR-11 as the essential viral miRNA and TFV-miR-13 and TFV-miR-14 as the specific miRNAs that contribute to HepG2 cell infection.

**Conclusions:**

We identified novel viral miRNAs and compared their expression in two host cells. The results of this study provide novel insights into the role of viral miRNAs in cross-species infection *in vitro*.

**Electronic supplementary material:**

The online version of this article (doi:10.1186/s12985-016-0530-6) contains supplementary material, which is available to authorized users.

## Background

As first identified in the nematode *Caenorhabditis elegans* [1, 2], microRNAs (miRNAs) have been found in almost all multicellular eukaryotes [3]. Since let7 from *C. elegans* was found completely conserved within the genomes of mice and humans [4], more than 15,000 novel miRNAs have been identified [5]. Mature miRNAs are noncoding RNAs approximately 21 to 22 nucleotides long [6] that regulate cellular processes such as immunity, apoptosis, development and other important aspects, by guiding the RNA-induced silencing complex to complementary mRNAs in 3’ untranslated regions (3’ UTRs) [7].

The first virus-encoded miRNAs were identified from Epstein–Barr virus (EBV) in 2004 [8]. Since then, several virus-encoded miRNAs have been found in different DNA viruses [9], including members of the *Herpesviridae*, *Polyomaviridae*, *Ascoviridae*, *Baculoviridae*, *Iridoviridae* and *Adenoviridae* families [10], and in a lone RNA virus, bovine leukemia virus [11]. The herpesviruses contribute the most virus-encoded miRNAs thus far and have been the focus of several studies on miRNA function [12]. Some virus-encoded miRNAs mimic host miRNAs and regulate the host transcripts to help the virus stay in the host [13]. For example, miR-BART5 encoded by EBV inhibits the protein p53 upregulated modulator of apoptosis (PUMA) to prevent cell apoptosis, which facilitates the establishment of latent infection [14]. Viral miRNAs also limit the lytic cycle; for instance, Kaposi’s sarcoma-associated herpesvirus (KSHV)-encoded miR-K12-1 targets the IκBα transcript 3’ UTR to reduce the expression and thus rescue the NF-κB activity inhibited by IκBα and delay viral lytic replication [15]. Virus-encoded miRNAs not only target cellular genes but also regulate viral genes. The KSHV miRNAs miR-K12-9 and miR-7-5p inhibit the expression of the replication and transcription activator (RTA), which is the master switch of the latent–lytic cycle [16, 17]. In Simian vacuolating virus 40, virus-encoded miRNAs cleave the early viral mRNAs to reduce the viral T-antigen expression at the late stages of infection and evade the immune response [18]. These studies show that virus-encoded miRNAs play vital roles during virus life cycle, which includes infection, replication, and release.

Iridoviruses are icosahedral cytoplasmic viruses that have double-stranded DNA genomes with terminal redundancy and circular permutation [19]. According to the Ninth Report of the International Committee on Taxonomy of Viruses, the *Iridoviridae* family consists of five genera: *Chloriridovirus*, *Iridovirus*, *Lymphocystivirus*, *Megalocytivirus* and *Ranavirus* [20]. Tiger Frog Virus (TFV) is the causative pathogen of abdominal distension disease, which causes a high mortality of tiger frog (*Rana tigrina rugulosa*) tadpoles cultured in Southern China [21]. TFV has been isolated from diseased tadpoles of tiger frog and classified as a member of the genus *Ranavirus* via complete genome sequencing [22]. Computational analysis revealed that the deduced TFV gene products exhibit more than 90 % similarity to those of frog virus 3 (FV3), the type species of *Ranavirus* [23]. As a member of *Ranavirus*, TFV can infect different cells, including fish cells, fathead minnow (FHM) cells, zebrafish embryonic fibroblast (ZF4) cells [24], and mammalian HepG2 cells [25], under laboratory conditions. The miRNAs encoded by iridoviruses were first identified using stem-loop quantitative real-time PCR (qRT-PCR) in Singapore grouper iridovirus (SGIV) [26]. In the present study, we identified novel viral miRNAs encoded by TFV and analyzed the diversity of miRNA expression in fish (ZF4) and mammalian (HepG2) cells to define the essential and specific miRNAs in cross-species *in vitro*.

## Methods

### Cells and virus

FHM cells were cultured in Medium 199 (Gibco, Life Technologies, USA) supplemented with 10 % fetal bovine serum (FBS) (Biochrom, Merck Millipore, Darmstadt, Germany) at 27 °C. ZF4 cells were cultured in Dulbecco’s Modified Eagle’s Medium/Nutrient Mixture F-12 (Gibco, Life Technologies, USA) supplemented with 10 % FBS at 27 °C. HepG2 cells were cultured in complete Dulbecco’s modified Eagle’s medium (Gibco, Life Technologies, USA) supplemented with 10 % FBS at 37 °C in a 2.5 % CO_2_-humidified chamber [27].

TFV was originally isolated from diseased tiger frog tadpoles from Nanhai (Guangdong, China) and is being maintained in our laboratory [22]. FHM cells were used as the host cells to propagate TFV at 27 °C, and the virus was harvested 3 days after the cytopathic effect was marked. Virus suspension was produced by freeze–thaw the virus–cell mixture three times, followed by filtration with a 0.45 μm Millex-HV Filter (Merck Millipore, Darmstadt, Germany). The virus titer was determined through the 50 % endpoint method using the 50 % tissue culture infective dose (TCID_50_), and a multiplicity of infection value of 10 was used for infection [28, 29].

### Sampling and RNA isolation

ZF4 and HepG2 cells were inoculated with TFV and incubated at 27 °C for 1 h. After infection, the inoculum was removed, and the two types of cells were cultured in their respective medium. Infected ZF4 cells were collected at 4, 12, 24, 48, and 72 h postinfection, whereas infected HepG2 cells were collected without medium at 4, 12, 24, 48, 72, 96, and 120 h postinfection. The two samples were then pooled for RNA isolation. Total RNA was extracted using TRI Reagent® (Sigma, USA) following the technical bulletin, quantified using an ND-1000 Nanodrop® Spectrophotometer (Thermo Scientific, USA), and then verified for quality using an Agilent 2100 Bioanalyzer (Agilent Technologies, USA). Only samples with an RNA integrity number greater than nine were used for sequencing.

### Small RNA library construction and Solexa sequencing

For small RNA library construction, approximately 20 μg of total RNA was size-fractionated on a denaturing polyacrylamide gel (PAGE), and small RNAs (18–30 nt) were eluted from the excised gel slice. Proprietary adaptors were then ligated to the 5’ and 3’ termini of the RNA to allow reverse transcription and PCR amplification. The generated cDNA library was used for Solexa sequencing in an Illumina Genome Analyzer (Illumina, USA) in accordance with the manufacturer’s instructions [30, 31].

### Sequencing data and annotation analysis

Sequencing data were cleaned by removing adaptor sequences, low-quality tags, and contaminants to obtain clean reads and assess the length distribution of small RNAs. After exploring small RNA distribution across the zebrafish (*Danio rerio*) and human (*Homo sapiens*) genomes, rRNA, tRNA, snRNA, snoRNA, and other noncoding RNAs were identified by alignment using the Rfam 10.1 (http://rfam.xfam.org/) and GenBank (http://www.ncbi.nlm.nih.gov/genbank/) databases; known miRNAs were identified by alignment using miRBase (Version 19.0) (http://www.mirbase.org/); and repeat-associated small RNAs and degradation fragments of mRNAs were identified.

### Novel miRNA identification

After annotation analysis, the unannotated sequences were aligned against the TFV genome (GenBank: AF389451.1) to search perfectly matched sequences for novel miRNA prediction. The 100 nucleotides flanking each side of the selected sequences were used to predict novel miRNA candidates by analyzing hairpin structures and calculating secondary structures with Mireap (http://sourceforge.net/projects/mireap/) [32]. Only sequences conserved within the stem loop that folded into hairpins were considered putative novel miRNAs.

### miRNA qRT-PCR

The infected cells were harvested, and total RNA was extracted using TRI Reagent® as described above. miRNA qRT-PCR primers specific for novel miRNAs and human U6 were designed by RiboBio (Guangzhou, China) and described as a bulge-loop™ miRNA qRT-PCR primer set (one reverse transcription primer and a pair of qRT-PCR primers in every set) [33]. The zebrafish U6 primers for signal normalization were designed as previously described [34]. Reverse transcription and RT-PCR were performed in LightCycler 480 (Roche, Mannheim, Germany) in accordance with the manufacturer’s instructions.

### Northern blot

Northern blot was performed as previously described with slight modifications [35]. In brief, 20 μg of total RNA extracted from infected cells was separated on a 15 % denaturing PAGE (7.5 M urea). The sample was transferred to positively charged nylon membranes (Roche, Mannheim, Germany) and cross-linked to the membranes through UV irradiation (120 mJ/cm^2^) [36]. After pre-hybridization in Ultrasensitive Hybridization Buffer (Ambion, Life technologies, USA) for more than 0.5 h at 37 °C, blots were hybridized overnight at 37 °C with 0.5 nM miRCURY LNA™ probes modified both by 5’ digoxigenin (DIG) and 3’ DIG (Exiqon, Vedbaek, Denmark). Afterward, the blots were sequentially washed using low-stringency, high-stringency, and washing buffers at 37 °C and then incubated in blocking buffer (Roche, Mannheim, Germany) for 3 h. The blots were subsequently incubated in blocking buffer with an anti-DIG-AP Fab fragment solution (Roche, Mannheim, Germany) for 0.5 h and then washed in DIG washing buffer (Roche, Mannheim, Germany). The blots were visualized through autoradiography using a CDP-Star chemiluminescent substrate for alkaline phosphatase (Roche, Mannheim, Germany).

### Transfection of the TFV miR-11 inhibitor

micr*OFF*™ inhibitor of TFV miR-11 and micr*OFF*™ inhibitor Negative Control #24 were purchased from RiboBio (Guangzhou, China). Lipofectamine® 2000 Transfection Reagent (Invitrogen, ThermoFisher Scientific, USA) was used for transfection to HepG2 in accordance with the standard protocol. In brief, the cells were seeded until 30 % confluence at transfection. The inhibitor (25 pmol) and Lipofectamine® 2000 Transfection Reagent (1 μL) were diluted in 50 μL of Opti-MEM® I Reduced Serum Medium (Invitrogen, ThermoFisher Scientific, USA) and then incubated for 5 min at room temperature. The inhibitor and Lipofectamine® 2000 Transfection Reagent were mixed and then incubated for 20 min at room temperature. The mixture was transferred to a 400 μL culture medium. After incubation at 37 °C for 4 h, the transfection mixture was replaced with fresh medium with 10 % serum and then infected with TFV.

### Absolute qRT-PCR of genomic DNA

The supernatant and the supernatant–cell mixture were harvested, and total genomic DNA was extracted using the DNeasy Blood & Tissue Kit (QIAGEN, USA). Absolute qRT-PCR was performed using primers for the TFV Major Caspd Protein (MCP) gene (forward primer: 5’-TCGCTGGTGGAGCCCTGGTA-3’, reverse primer: 5’-GGCGTTGGTCAGTCTGCCGTA-3’). This primer pair was used to amplify a region from 97034 to 97163 of the TFV genome. The plasmid pCMV-myc-TFV MCP, which served as the internal standard, was serially diluted by 10-fold to generate a standard curve of absolute qRT-PCR. The PCR reaction contained 5 μL of 2 × SYBR® Premix Ex Taq™ (TaKaRa, China), 1 μL of DNA template, 0.2 μL of 10 μM primers, and 3.6 μL of H_2_O. The absolute qPCR conditions were as follows: one cycle of 95 °C for 20 s and 40 cycles of 10 s at 95 °C, 20 s at 60 °C, and 1 s at 72 °C. Absolute qRT-PCR was performed at three replicates per sample.

This study was performed in strict accordance with the recommendations in the Guide for the Institutional Animal Care and Use Commission (IACUC). The protocol was approved by the Committee on the Ethics of Animal Experiments of the Sun Yat-sen University. All surgery was performed under MS-222 anesthesia, and every effort was made to minimize suffering.

## Results

### Deep sequencing of small RNAs from ZF4 and HepG2 cells infected by TFV

**Fig. 1:**
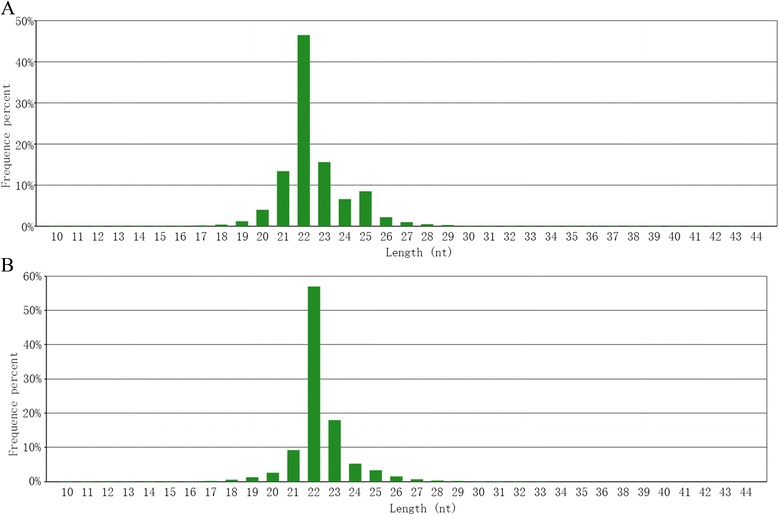
Length distribution of small RNAs derived from TFV-infected HepG2 and ZF4 cells. **a** Small RNAs derived from HepG2 cells; **b** small RNAs derived from ZF4 cells. The size of small RNAs ranges from 20 nt to 25 nt, and 22 nt reads are the most abundant, which is suitable for miRNA analysis

From the deep sequencing raw data of small RNAs derived from HepG2 (Group H) and ZF4 (Group Z) cells infected with TFV, we obtained 22,095,484 reads from Group H, including 22,030,626 high-quality reads, and 26,116,906 reads from Group Z, including 25,952,342 high-quality reads. After removing adaptors, 21,850,841 and 25,718,113 clean reads covering 99.18 and 99.10 % of the total reads from Groups H and Z, respectively, were annotated and analyzed. The length distribution mainly covered 20–25 nt, and 22 nt reads were the most abundant, which was suitable for miRNA analysis (Fig. 1). The clean reads that were mapped to the human and zebrafish genomes were aligned to the miRNA precursors of human and zebrafish in miRBase (Versioin 19.0) to obtain the known miRNA count. The clean reads were also aligned to repeat-associated RNAs to find matched reads and were annotated with rRNA, scRNA, snRNA, snoRNA, srpRNA, and tRNA from Rfam (Table 1).

**Table 1:** Distribution of small RNAs from TFV-infected HepG2 and ZF4 cells

	Group H^a^		Group Z^a^	
	Total sRNAs	Percentage (%)	Total sRNAs	Percentage (%)
total reads	21850841	100 %	18247263	70.95 %
miRNA	9692020	44.36 %	1356769	5.28 %
rRNA	8055249	36.86 %	721113	2.80 %
scRNA	73186	0.33 %	594	0.00 %
snRNA	134343	0.61 %	80205	0.31 %
snoRNA	56098	0.26 %	44001	0.17 %
srpRNA	1027	0.00 %	0	0.00 %
tRNA	346129	1.58 %	669094	2.60 %
unannotation^b^	3372212	15.43 %	4437921	17.26 %

^a^Group H and Group Z refer to the sample from infected HepG2 and ZF4 cells

^b^unannotation indicates the reads do not match the annotated RNA databases and human and zebrafish genome

### Prediction of novel TFV-encoded miRNAs

**Fig. 2:**
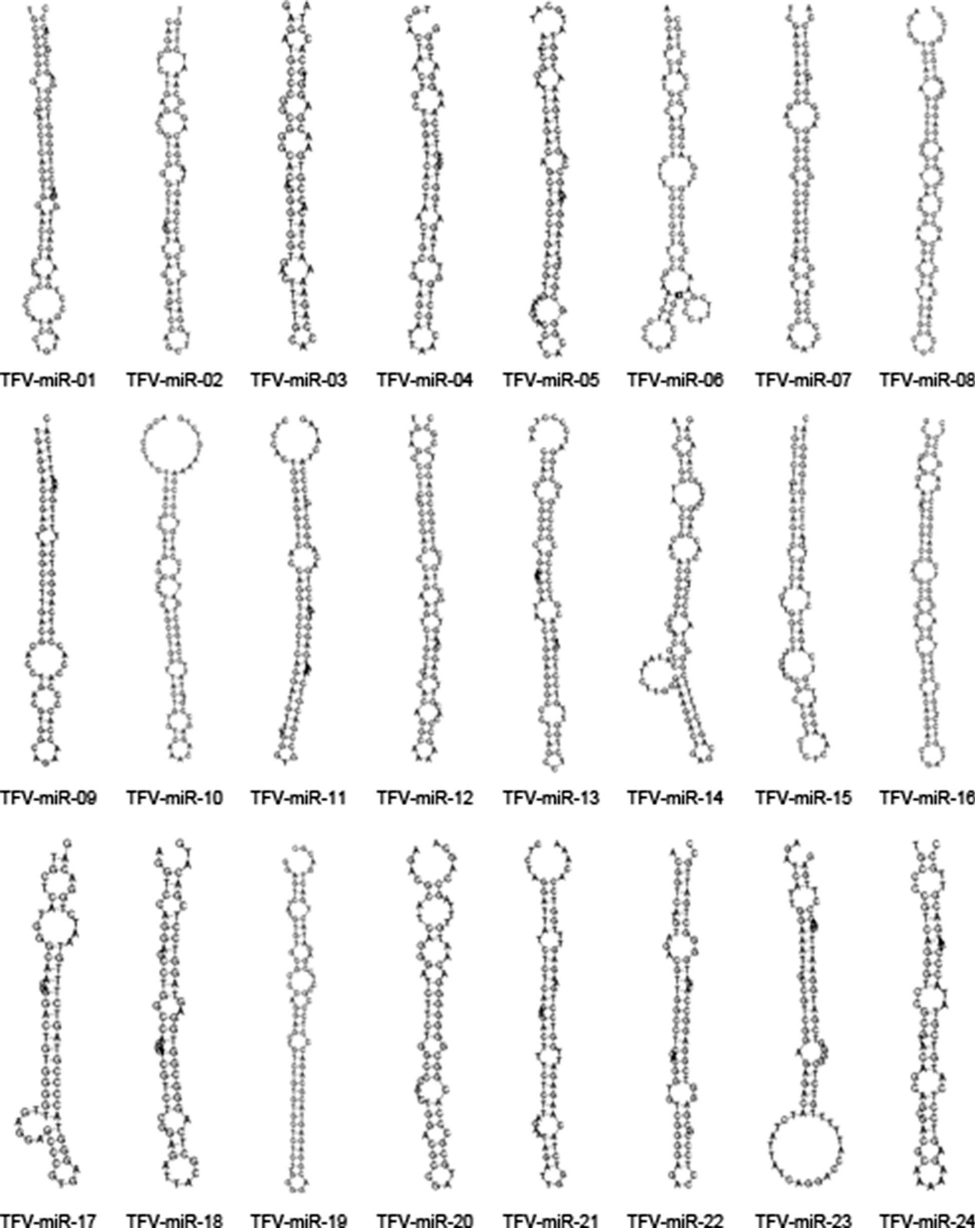
RNA folding structure of predicted novel TFV-encoded miRNAs

Two groups of clean reads were aligned to the TFV genome and then aligned against known virus-encoded miRNAs at miRBase (Version 19.0), after which 71,342 and 805,460 reads of small RNAs were found from Groups H and Z, respectively (Table 2). The reads that perfectly matched the TFV genome but not found at miRBase (Versioin 19.0) were considered to explore the hairpin structure of the miRNA precursor. The structure stability of each novel miRNA precursor was analyzed using Mireap (http://sourceforge.net/projects/mireap/), and all the candidate miRNAs were folded to a stable hairpin structure (Fig. 2). Mireap is used to predict novel miRNAs by exploring the secondary structure, the Dicer cleavage site and the minimum free energy of the unannotated small RNA tags that could be mapped to the TFV genome. Novel TFV-encoded miRNAs were predicted using Mireap, and 24 novel miRNAs precursors were identified to generate 24 mature miRNAs. The names and sequences of these miRNAs are listed in Table 3.

**Table 2:** Small RNAs matched to TFV genome

	Group H		Group Z	
	Total sRNAs	Percentage (%)	Total sRNAs	Percentage (%)
total reads	21850841	100 %	18247263	70.95 %
Known miRNA	71342	0.33 %	805460	3.13 %
miRNA matched to TFV genome	386933	1.77 %	865830	3.37 %

**Table 3:** Names and sequences of TFV-encoded miRNAs

Name	Sequence	Length
TFV-miR-01	5’- GTTGCGGTGCCTGGGGTCGGGA -3’	26
TFV-miR-02	5’- AGACGGTCGGGCTTGACGTTGAG -3’	27
TFV-miR-03	5’- GCGGGCACAGGGTGGTGACTTT -3’	26
TFV-miR-04	5’- GCTGGTGTAGATGGTGCCTGTCC -3’	27
TFV-miR-05	5’- AGACAGCTGCCTGACGTGAAA -3’	25
TFV-miR-06	5’- AGGCGGTGGCGTCGTAGGTCTTG -3’	27
TFV-miR-07	5’- GGACCTGCCGTCGGGGACGTG -3’	25
TFV-miR-08	5’- AGTCTCGGGCCTGAAGGGAAGGAC -3’	28
TFV-miR-09	5’- GTAGGCCTTGACGCACCTGACGTG -3’	28
TFV-miR-10	5’- AGGCTGATGTCCATGTCGTCG -3’	25
TFV-miR-11	5’- TGAGGGTGACCTGACAGGGCTGT -3’	27
TFV-miR-12	5’- GAGCCAAGTCTGCCTGTCGTCGGC -3’	28
TFV-miR-13	5’- GGACGTCCCCGCGCCCGTGCTG -3’	26
TFV-miR-14	5’- CCTGCACACGGGGTCGGACGCCA -3’	27
TFV-miR-15	5’- GAGTCTCTCTTGTTGGTCTTTGG -3’	27
TFV-miR-16	5’- CCCACTGGACGGGCTCGGACGGCC -3’	28
TFV-miR-17	5’- CAACGGGACTGTGGGGTTGAG -3’	25
TFV-miR-18	5’- GCTCAGGGCGGTGGAGTAGGT -3’	25
TFV-miR-19	5’- TGTGCGCCCAGGAGCTGTCTGT -3’	26
TFV-miR-20	5’- CCCACGGCGGGGGGGACAATGT -3’	26
TFV-miR-21	5’- AGAGATTGGTCCTGAAGAGTTT -3’	26
TFV-miR-22	5’- AGACGTTGGCCTCGACGGTGTCGG -3’	28
TFV-miR-23	5’- TCTCGGGGTCGATGGAATTTG -3’	25
TFV-miR-24	5’- GGGTCCGCGTACAGCAGCGACG -3’	26

### Localization of TFV-encoded miRNAs in genome

**Fig. 3:**
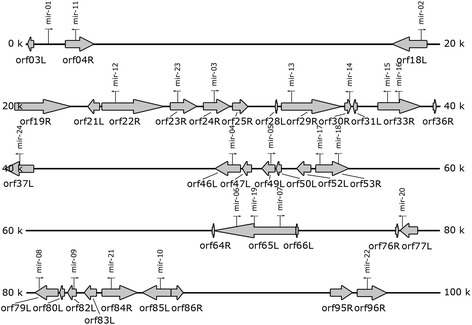
Localization of predicted miRNAs in the TFV genome. The location, size, and transcriptional orientation of ORFs close to the predicted miRNAs are indicated by large arrows, and the predicted TFV-encoded miRNAs are indicated by small arrows. TFV-miR-02, -04, -05, -06, -07, -08, -10, -11, -12, -13, -14, -15, -16, -17, -18, -21, -22, and -23 are antisense to those located within ORF, whereas TFV-miR-05, -07, -08, -09, -14, -15, -16, -19, -20, -22, -23, and -24 are located in the 3’ downstream sequence of the closest ORF

All predicted novel TFV-encoded miRNAs were widely distributed throughout the TFV genome (Fig. 3). Almost all of the TFV-encoded miRNAs were located within the TFV open reading frames (ORFs), except TFV-miR-01, which was located in the intragenic region between the 5’ upstream sequence of ORF003L and ORF004R. The miRNAs TFV-miR-15 and -16, TFV-miR-17 and -18, as well as TFV-miR-06, -07, and -19, shared the same ORFs. The miRNAs showed two types of miRNA localization on the basis of the transcriptional orientation. In the first type, the miRNAs, including TFV-miR-02, -04, -05, -06, -07, -08, -10, -11, -12, -13, -14, -15, -16, -17, -18, -21, -22, and -23, were antisense to the location within the ORF. In the second type, the miRNAs, including TFV-miR-05, -07, -08, -09, -14, -15, -16, -19, -20, -22, -23, and -24, were located in the 3’ downstream sequence of the neighboring ORF. These two types of miRNAs covered 22 out of the 24 predicted novel TFV-encoded miRNAs and may be potential modulators of the virus genes [12].

### Verification of novel TFV-encoded miRNAs by bulge-loop qRT-PCR

**Fig. 4:**
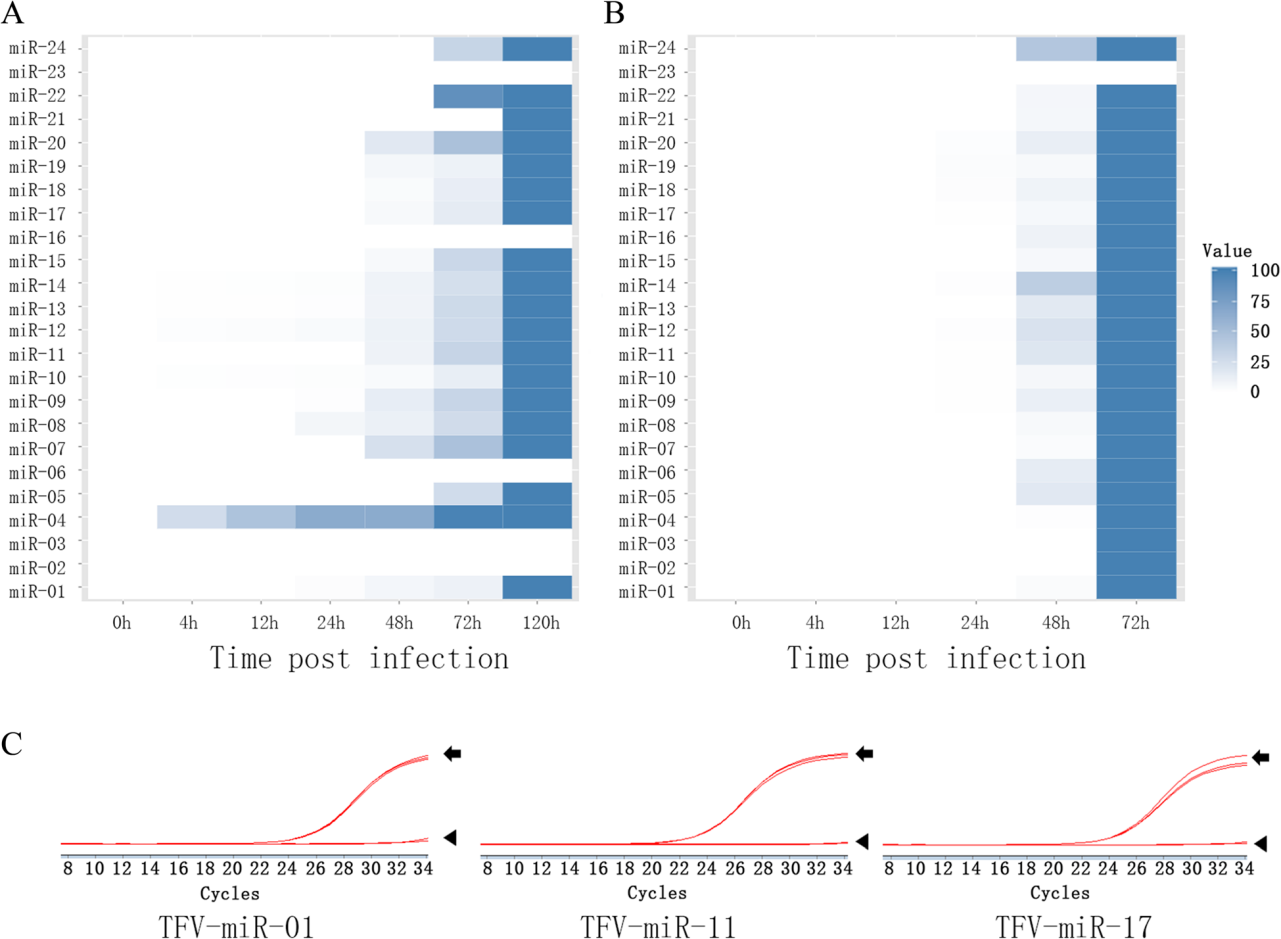
Verification of TFV-encoded miRNAs from infected HepG2 and ZF4 cells by bulge-loop qRT-PCR. **a** Expression profiles of miRNAs from HepG2 cells infected by TFV. TFV-miR-01, -04, -05, -07, -08, -09, -10, -11, -12, -13, -14, -15, -17, -18, -19, -20, -21, -22, and -24 were detected in Group H. **b** Expression profiles of miRNAs from ZF4 cells infected by TFV. TFV-miR-01, -02, -03, -04, -05, -06, -07, -08, -09, -10, -11, -12, -13, -14, -15, -16, -17, -18, -19, -20, -21, -22, and -24 were detected in Group Z. **a**, **b** Expression of miRNAs increased with the period of infection. **c** Amplification curves of qRT-PCR. Compared with the uninfected cells, the typical miRNAs (TFV-miR-01, -11, and -17) detected by qRT-PCR show notable amplification curves. The black arrow indicate the curve of miRNAs, the black arrow head indicate the curve of negative control that is from uninfected cells

Bulge-loop qRT-PCR was used to detect the expression of mature miRNAs and thus verify the novel TFV-encoded miRNAs. Human and zebrafish U6 were used to normalize the data, and uninfected HepG2 and ZF4 cells were considered as negative controls for Groups Z and H, respectively. The highest expression level of each miRNA was defined as the 100-value, and the expression levels at other time points were calculated relative to the highest one. Cycle thresholds greater than 30 were considered negative to identify miRNAs based on qRT-PCR data [37, 38]. The following 19 and 23 miRNAs were detected in Groups H and Z, respectively: TFV-miR-01, -04, -05, -07, -08, -09, -10, -11, -12, -13, -14, -15, -17, -18, -19, -20, -21, -22, and -24 in Group H, and TFV-miR-01, -02, -03, -04, -05, -06, -07, -08, -09, -10, -11, -12, -13, -14, -15, -16, -17, -18, -19, -20, -21, -22, and -24 in Group Z. The predicted TFV-miR-23 was undetected in both groups. Considering that qRT-PCR is a highly sensitive method to detect RNA expression, we supposed that TFV-miR-23 is nonexistent (Fig. 4a, Additional file 1: Figure S1).

In both groups, miRNA expression increased with the period of infection. In Group H, TFV-miR-4, -10, -12, -13, and -14 were detected at 4 h postinfection (hpi); TFV-miR-01, -08, and -09 were detected at 24 hpi; TFV-miR-07, -11, -15, -17, -18, -19, and -20 started at 48 hpi; and TFV-miR-05, -22, and -24 were expressed last at 72 hpi. For Group Z, no miRNAs were detected at 4 hpi; the earliest miRNAs appeared at 24 hpi, including TFV-miR-09, -10, -11, -12, -14, -17, -18, -19, and -20; TFV-miR-01, -04, -05, -06, -07, -08, -13, -15, -16, -21, -22, and -24 were detected at 48 hpi; and TFV-miR-2 and -3 appeared at 72 hpi (Fig. 4b, Additional file 2: Figure S2). The amplification curves of some typical miRNAs between the highest expression and negative control (uninfected cells) are shown in Fig. 4c. These results verified existing miRNAs and showed that viral miRNAs were expressed earlier in Group H, i.e., the infected mammalian cells, than in Group Z.

### Northern blot of TFV-encoded miRNAs

**Fig. 5:**
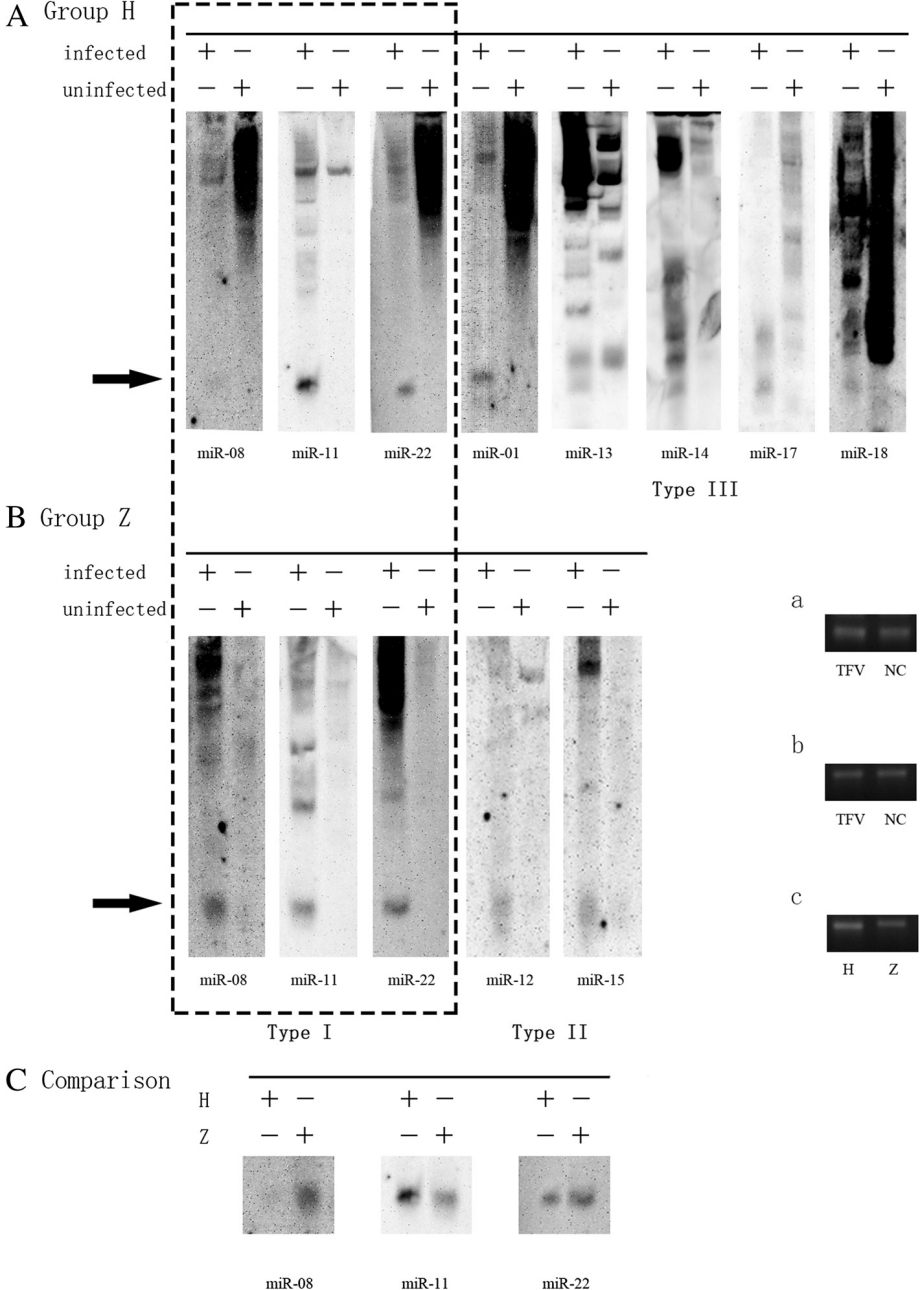
Northern blot results of TFV-encoded miRNAs from infected HepG2 (Group H) and ZF4 (Group Z) cells. **a** TFV-miR-01, -11, -17, -18, and -22 were detected in Group H. **b** TFV-miR-01, -04, -08, -11, -12, -15, and -22 were detected in Group Z. **c** Comparison of TFV-miR-08, -11, and -22 between Groups H and Z; *a* 18S RNA of HepG2 cells; *b* 18S RNA of ZF4 cells; *c* 18S RNA of HepG2 and ZF4 cells. TFV-miR-08 and -22 had higher expression levels in Group Z, whereas TFV-miR-11 had higher expression levels in Group H. Type I miRNAs can be detected in both groups and Type II and Type III miRNAs can only be detected in Groups H and Z respectively. The black arrow indicates the positions of mature miRNAs; H, HepG2 cells; Z, ZF4 cells. Dashed box indicates the Type I miRNAs detected in Groups H and Z

We used the gold standard, Northern blot, to detect and validate the TFV-encoded miRNAs. The expression profiles helped us choose the optimal times of miRNA expression. These times of expression, which were positively verified via qRT-PCR, should also be validated by Northern blot. Uninfected HepG2 and ZF4 cells were considered as negative controls for Groups H and Z, respectively. TFV-miR-11 mimic was taken as a positive control to be ran at every gel to determine the size of mature miRNAs (Additional file 3: Figure S3). Three types of miRNAs were revealed by the Northern blot results. Type I includes the miRNAs that were expressed in both groups, including TFV-miR-08, -11, and -22 (Fig. 5a and b). Type II includes the miRNAs that were detected only in Group H, including TFV-miR-01, -13, -14, -17, and -18 (Fig. 5a). Type II includes the miRNAs that were detected only in Group Z, including TFV-miR-12 and -15. (Fig. 5b). Considering that these miRNAs can also be detected through qRT-PCR, we determined that TFV-miR-01, -08, -11, -12, -13, -14, -15, -17, -18, and -22 were indeed viral miRNAs. Given its abundance in both groups as validated by Northern blot, TFV-miR-11 was defined as the essential viral miRNA.

### Comparison of expression profiles of TFV-encoded miRNAs in infected HepG2 and ZF4 cells

**Fig. 6:**
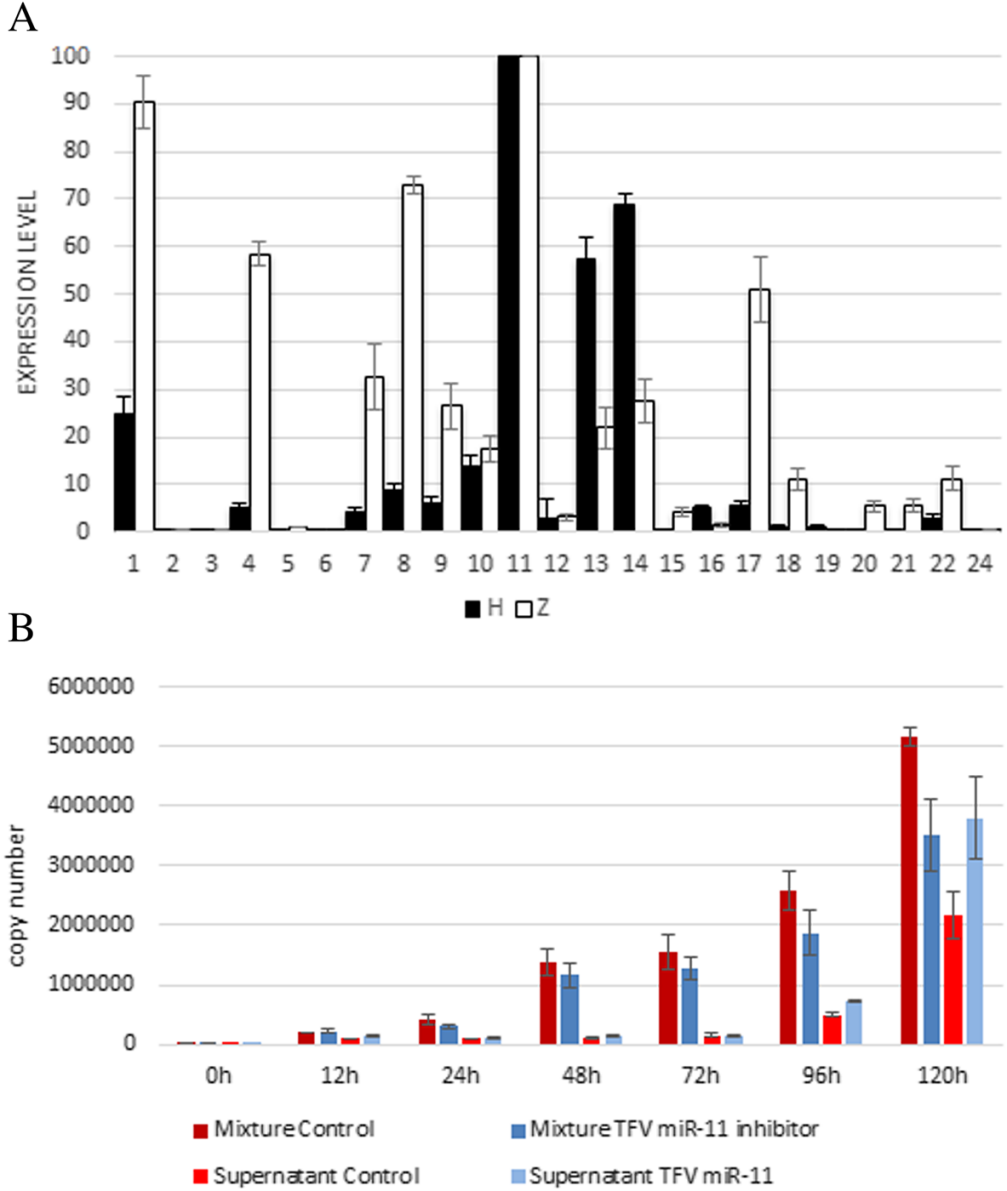
**a** Difference in the expression of TFV-encoded miRNAs between infected HepG2 (Group H) and ZF4 (Group Z) cells. As the most abundant miRNA, TFV-miR-11 was taken as the 100-value, with the expression levels of all the other novel miRNAs calculated relative to it. Most of the miRNAs had higher expression levels in Group Z, except for TFV-miR-13 and -14, which were more highly expressed in Group H. **b** Role of TFV miR-11 in TFV life cycle. Absolute qRT-PCR was applied to detect the copy number of virions by amplifying MCP. The TFV miR-11 inhibitor and negative control were transfected HepG2 cells following TFV infection. The samples were harvested at 12, 24, 48, 72, 96, and 120 h post-infection. Mixture: mixture of cells and supernatant; supernatant: supernatant from infected cells

TFV can infect a wide range of host cells, including fish and mammalian cells, under laboratory conditions. However, the mechanism underlying cross-species infection remains unclear. We analyzed the role of miRNAs encoded by TFV in such cross-species infections. Considering that HepG2 and ZF4 cells have been reported as the respective mammalian and fish infection models for TFV, we further compared the expression levels of miRNAs in these two groups. As described above, the peak expression of all the miRNAs was observed at 120 and 72 hpi in Groups H and Z, respectively. Thus, all of the expression levels of miRNAs were compared at 120 and 72 hpi between Groups H and Z, respectively. The most highly expressed miRNA in both groups was TFV-miR-11, as validated by Northern blot, and the copy numbers of TFV-miR-11 were 7 × 10^9^ in Group H and 1.11 × 10^9^ in Group Z by absolute qRT-PCR. TFV miR-11 mimic was used as the internal standard and was serially diluted by 10-folds to generate a standard curve following the standard miRNA qRT-PCR (Additional file 4: Figure S4). Hence, we considered TFV-miR-11 as the 100-value standard to which other miRNAs in the same group were compared. As shown in Fig. 6, qRT-PCR results revealed that most of the miRNAs had higher expression levels in Group Z, except for TFV-miR-13 and -14, which had higher expression levels in Group H. Interestingly, TFV-miR-13 and -14 were only detected in Group H via Northern blot (Fig. 5a). Thus, we defined them as specific viral miRNAs in HepG2 cells that may play specific roles during mammalian cell infection.

We also compared the expression levels of TFV miR-08, -11, and -22 by performing Northern blot, with 18S RNA as the control. Consistent with the results of qRT-PCR, TFV-miR-08 and -22 had higher expression levels in Group Z, whereas TFV-miR-11 had a higher expression level in Group H (Fig. 5c). The difference in expression levels of the TFV-encoded miRNAs from the two groups might provide a clue to elucidate the mechanism by which the virus adapts itself to different types of host cells.

### TFV miR-11 antagomir inhibits the production and induces the release of TFV virions

TFV miR-11 is the most abundant miRNA among all the TFV encoded miRNAs in both Groups H and Z. We transfected the TFV miR-11 inhibitor by binding to mature miRNAs into HepG2 cells following TFV infection to test the role of TFV miR-11 in the life cycle of TFV. We detected the amount of virions from the supernatant and the supernatant–cell mixture through absolute qRT-PCR of TFV MCP. In the series of infection time points, viral genomic DNA was detected at 12 hpi and significantly increased at 48 hpi in the supernatant–cell mixture. Less viral genomic DNA was detected in the TFV miR-11 inhibitor-transfected cells at every time point (Fig. 6b). A large amount of viral genomic DNA was detected at 96 hpi in the supernatant, and much more viral genomic DNA was detected after transfection with the TFV miR-11 inhibitor (Fig. 6b). At 120 hpi, the copy numbers of viral genomic DNA in the supernatant and the mixture were almost the same. This result indicates that almost all the virions were released from the hosts after transfection with TFV miR-11. This result indicates that TFV miR-11 induces virion release from host cells.

## Discussion and Conclusions

Several studies have identified miRNAs encoded by DNA viruses, but little is known about the roles of viral miRNAs in cross-species infection. As a member of the genus *Ranavirus* [22], TFV can infect a wide range of host cells under laboratory conditions, similar to the type species FV3; thus, TFV can be used as a model to investigate the functions of miRNAs in different types of host cells [24, 25, 39]. In the present study, we selected HepG2 and ZF4 cells as infection models to investigate the distribution of miRNA expression in cross-species infection. After deep sequencing small RNAs, we obtained 22,030,626 and 25,952,342 high-quality reads from infected HepG2 and ZF4 cells, respectively, and predicted 24 novel viral miRNAs. We then identified 23 novel TFV-encoded miRNAs through qRT-PCR and validated 10 of them through Northern blot. This study is the first to report the diversity of miRNA expression in TFV-infected HepG2 and ZF4 cells and define TFV-miR-11 as the essential viral miRNA and TFV-miR-13 and -14 as the specific viral miRNAs for HepG2 infection. We transfected the TFV miR-11 inhibitor before infection and found that this inhibitor induce the release of virions. Significant differences in miRNA expression were found between HepG2 and ZF4 cells, indicating new mechanisms of cross-species infection.

The patterns of viral miRNA location in the genome significantly varied among different types of viruses, which provides clues to the function of viral miRNAs [12]. The 12 miRNAs encoded by KSHV, a well-known oncogenic human herpesvirus, were located as a cluster in the region of ORF K12 mRNA to the start codon of ORF71 [40, 41]. These 12 miRNAs are highly expressed in latently infected B cells, miR-K12 is located in ORF K12, miR-K10 is located in the 3’ UTR of K12, and the 10 other miRNAs are all in one intron, which is reminiscent of the function of latent infection by KSHV-encoded miRNAs [17]. For the first viral miRNAs identified in iridovirus, the locations of SGIV-encoded miRNAs are completely different. The SGIV-encoded miRNAs are scattered throughout the entire genome, situated both on and in between the ORFs. This type of miRNA may not only focus on one virus gene but also on a number of genes from both the virus and the host. Similar to SGIV-encoded miRNAs, TFV-encoded miRNAs are located throughout the entire genome. Those that are antisense to the ones located within ORF and in the 3’ downstream sequence of the neighboring ORFs may inhibit the corresponding viral genes. Others such as TFV-miR-01 located in the intragenic region between the 5’ upstream sequences of ORF003L and ORF004R may focus on host mRNAs.

The aim of our future work is to reveal the functions of TFV-encoded miRNAs. To date, the functions of virus-encoded miRNA can be classified into two groups: one is to mimic the host miRNA by sharing the same seed region to modulate the target mRNA, and the other is to target viral genes. As mentioned above, the localization of TFV-encoded miRNAs in the genome revealed the potential function specific to the viral genes. The regulation of viral protein expression by the miRNA encoded by itself slightly differ from the common pattern [42]. Aside from targeting the 3’ UTR of the viral transcripts [16, 17], virus-encoded miRNAs can also bind viral mRNAs perfectly matched for degradation [18, 43], and the target binding site is also located within the 5’ UTR of the mRNA [44]. In the present study, we characterized the localization of each miRNA in the genome and found that TFV-miR-22 was located in TFV ORF096R and antisense to the transcript. Previous reports have verified ORF096R as the major capsid protein [45], which is the basic structural protein of the virus. Another miRNA that drew our interest is TFV-miR-08, which was detected by Northern blot in both groups, located in the opposite strand of the 3’ downstream sequence of TFV ORF080L. ORF080L is a putative membrane-associated motif in LPS-induced tumor necrosis factor alpha factor (LITAF), a transcription factor affecting TFV-α expression [46] and regulating inflammatory cytokines [47]. Recent investigations have revealed that LITAF colocalizes with FV3 ORF75L [48], which is highly similar to TFV ORF080L, and that SGIV-miR-13 is located in SGIV ORF136R, which has been designated as LITAF [26]. Although the 3’ UTR of TFV ORF096R has not been identified, recent investigations on LITAF have motivated us to seek the relationship between TFV-miR-08 and LITAF in terms of virus pathogenesis. Interestingly, TFV-miR-18 shares the same seed as human miR-1266. Although its seed region is the same as human miRNA and it can be detected in Group H by Northern blot, TFV-miR-18 may be capable of regulating human genes that may be key for TFV infecting mammalian cells.

Similar to FV3, TFV has a wide range of hosts under laboratory conditions, and its pathogenesis in cross-species infections is intriguing [49]. In addition, the miRNA expression pattern in different host cells provides novel insights into the pathogenesis of TFV. The qRT-PCR results described a complete view of all the miRNAs, showing that TFV-miR-11 is the most abundant among the miRNAs. The diversity of TFV-miR-08, -11, and -22 correlated with the Northern blot result that TFV-miR-08 and -22 had higher expression levels in Group Z and TFV-miR-11 had a higher expression level in Group H. Considering that TFV-miR-11 had the highest expression levels in both groups and can be detected by both methods, we conclude that TFV-miR-11 is the essential viral miRNA for TFV life cycle and that TFV-miR-08 and -22 are important in maintaining TFV in different host cells. Moreover, TFV-miR-13 and -14 levels significantly increased in group H and were only detected in group H by Northern blot. This result suggests that these two miRNAs have specific roles in the TFV infection of mammalian cells. However, the mechanism and function of these miRNAs warrant further investigation.

## Additional files

Additional file 1: Figure S1. Expression profiles of miRNAs from HepG2 cells infected by TFV. (TIF 181278 kb) Additional file 2: Figure S2. Expression profiles of miRNAs from ZF4 cells infected by TFV. (TIF 181277 kb) Additional file 3: Figure S3. Original images of Northern blots. I. infected cells; U. uninfected cells. (TIF 26598 kb) Additional file 4: Figure S4. Standard Curve of TFV miR-11. (TIF 712 kb)
