# Supplementary figures and images for: Identification and differential expression analysis of MicroRNAs encoded by Tiger Frog Virus in cross-species infection in vitro
Source: Virol J. 2016 Apr 30;13:73. doi: 10.1186/s12985-016-0530-6 (PMC4851794; doi:10.1186/s12985-016-0530-6)

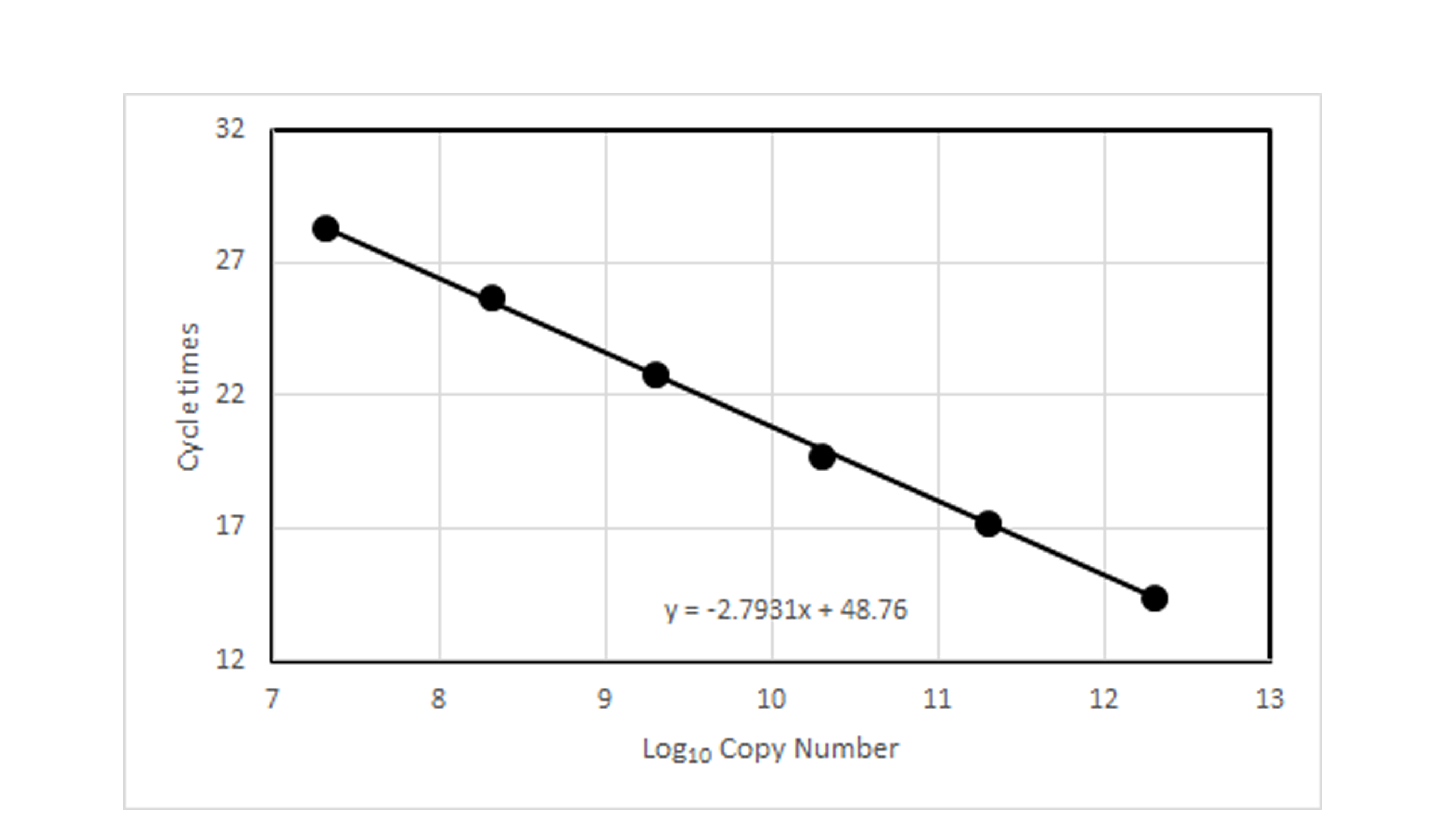

Supplement: Additional file 4: Figure S4. — Standard Curve of TFV miR-11. (TIF 712 kb) [file 12985_2016_530_MOESM4_ESM.tif]
